# Before the bolus: why anti-amyloid therapy must become part of every stroke code

**DOI:** 10.1097/MS9.0000000000005585

**Published:** 2026-08-21

**Authors:** Abdul S. Khaliq, Kaleem Khan

**Affiliations:** a Trauma and Emergency Department, Jinnah Hospital, Lahore, Pakistan; b Department of Surgery, Balkh University, Mazar-i-Sharif, Balkh, Afghanistan

**Keywords:** acute ischemic stroke, amyloid-related imaging abnormality, anti-amyloid immunotherapy, intravenous thrombolysis, mechanical thrombectomy

## Abstract

Anti-amyloid monoclonal antibodies provide disease-modifying treatment options for selected patients with early symptomatic Alzheimer disease, but introduce new challenges for acute stroke care. Amyloid-related imaging abnormalities (ARIA) are often asymptomatic; however, symptomatic ARIA may cause aphasia, focal weakness, visual disturbance, confusion, headache, or seizures, and can resemble acute ischemic stroke. Initial noncontrast computed tomography may be nondiagnostic in both conditions, and failure to identify anti-amyloid exposure can complicate time-sensitive reperfusion decisions. Current regulatory guidance advises caution when considering thrombolytic therapy because serious and fatal intracerebral hemorrhage has been reported, although the absolute risk and a safe interval following anti-amyloid administration remain uncertain. Conversely, attributing every acute neurological deficit to ARIA could delay effective reperfusion for a true ischemic stroke. This editorial examines the clinical and imaging features relevant to this diagnostic overlap and proposes a systems-based response involving reliable medication identification, rapid vascular imaging, targeted magnetic resonance imaging, specialist collaboration, and separate consideration of intravenous thrombolysis and mechanical thrombectomy. Anti-amyloid exposure should be recognized as time-critical information during stroke evaluation. The objective is not automatic exclusion from reperfusion, but an individualized approach that minimizes avoidable hemorrhagic harm while preserving appropriate treatment for confirmed cerebral ischemia.

## Introduction

The introduction of anti-amyloid monoclonal antibodies, including lecanemab and donanemab, has changed the treatment landscape for selected patients with early symptomatic Alzheimer disease while creating new challenges for acute neurological care. These therapies are associated with amyloid-related imaging abnormalities (ARIA), which include vasogenic edema or sulcal effusion (ARIA-E) and cerebral microhemorrhage, superficial siderosis, or larger intracerebral hemorrhage (ARIA-H)[1]. Although most radiographically detected events are asymptomatic, symptomatic ARIA may present with aphasia, focal weakness, visual disturbance, confusion, headache, or seizures. This clinical spectrum can resemble acute ischemic stroke, particularly when symptoms develop abruptly. Noncontrast computed tomography can identify an established intracranial hemorrhage but may be normal or nondiagnostic in early ischemia and ARIA, creating substantial uncertainty during a time-sensitive stroke evaluation[2].

Failure to identify recent anti-amyloid exposure may have important consequences because thrombolytic therapy in the presence of treatment-related vascular vulnerability could increase the risk of intracerebral bleeding. Conversely, attributing every acute neurological deficit in a treated patient to ARIA could delay effective reperfusion for a true ischemic stroke. The objective of this editorial is to examine this emerging diagnostic and therapeutic intersection, clarify the clinical and imaging considerations that may help distinguish symptomatic ARIA from acute ischemic stroke, and propose a practical systems-based approach to medication recognition, urgent imaging, and individualized reperfusion assessment. By connecting memory-clinic prescribing with emergency stroke pathways, this work aims to support safer decision-making without allowing appropriate caution to result in therapeutic delay or inappropriate exclusion from reperfusion.

## The stroke code has changed

The frequency of ARIA in pivotal trials demonstrates why anti-amyloid exposure has become relevant to acute stroke assessment. In CLARITY AD, ARIA-E occurred in 12.6% of lecanemab-treated participants and was symptomatic in 2.8%[3]. In TRAILBLAZER-ALZ 2, ARIA-E occurred in 24.0% of donanemab-treated participants, with symptomatic events reported in 52 participants, corresponding to approximately 6.1%[4]. These estimates should not be compared directly because the trials differed in their populations, treatment protocols, dosing schedules, and imaging requirements. Nevertheless, they establish symptomatic ARIA as a recognized treatment-related safety concern rather than a purely theoretical complication.

This concern extends beyond memory clinics because symptomatic ARIA may first become apparent during an emergency stroke evaluation. The 2025 American Heart Association science advisory emphasized that ARIA-related symptoms can resemble acute ischemic stroke and highlighted the expanding role of vascular neurologists in caring for patients receiving anti-amyloid immunotherapy[5]. Stroke teams should therefore recognize anti-amyloid exposure as time-critical medication information, similar in urgency to anticoagulant use. The challenge is not simply to detect ARIA but to determine whether an acute neurological deficit represents ARIA, cerebral ischemia, intracranial hemorrhage, seizure-related dysfunction, or multiple coexisting processes.

## Understanding symptomatic ARIA

ARIA are conventionally classified as ARIA-E, which includes vasogenic edema and sulcal effusion, and ARIA-H, which includes cerebral microhemorrhage, superficial siderosis, and larger intracerebral hemorrhage. These abnormalities are thought to arise partly from the mobilization of amyloid through cerebral vessels that may already be affected by cerebral amyloid angiopathy. Risk is not uniform and is generally higher early in treatment, among apolipoprotein E (APOE) ε4 carriers, particularly homozygotes, and in patients with a greater baseline burden of microhemorrhage or superficial siderosis[6]. The specific treatment agent, dosing schedule, blood pressure, and exposure to antithrombotic medications may also influence risk, although several of these relationships remain incompletely characterized.

Most radiographically detected ARIA are asymptomatic, and many events resolve with treatment interruption and appropriate monitoring. Symptomatic cases may present with headache, confusion, aphasia, focal weakness, visual disturbances, gait impairment, nausea, or seizures. A 2025 secondary analysis of the donanemab trials found that headache and confusion were among the most frequently reported symptoms, while serious or severe events occurred less frequently. Clinical severity may not always correspond closely to the initial radiographic burden[7]. Recent treatment exposure, accompanying headache or encephalopathy, seizure activity, and fluctuating or progressive deficits should increase suspicion for ARIA. However, none of these features can safely exclude acute cerebral ischemia. Sudden focal symptoms in a treated patient must therefore continue to trigger an urgent stroke evaluation.

## The diagnostic collision

The principal diagnostic difficulty is that early ischemic stroke and symptomatic ARIA can overlap clinically and may not be reliably differentiated by initial noncontrast computed tomography (CT). Noncontrast CT can identify an established macrohemorrhage but does not reliably exclude early infarction, ARIA-E, cerebral microhemorrhage, or superficial siderosis. Treatment history should therefore be obtained in parallel with the standard stroke assessment, including the specific anti-amyloid agent, date of the most recent dose, previous ARIA events, baseline magnetic resonance imaging (MRI) findings, APOE genotype when known, and current antithrombotic treatment^[^5,8^]^. This information should support, rather than delay, urgent neurological examination and imaging.

CT angiography is particularly important because demonstration of an arterial occlusion provides evidence of a potentially treatable ischemic mechanism. Conversely, the absence of an explanatory occlusion does not establish ARIA because small-vessel ischemia and other stroke mimics remain possible[9]. When immediately available and likely to alter management, MRI offers greater diagnostic resolution. Diffusion-weighted imaging can support the diagnosis of acute infarction by demonstrating restricted diffusion. Fluid-attenuated inversion recovery sequences can identify vasogenic edema or sulcal effusion, while susceptibility-weighted or gradient-echo sequences can detect microhemorrhage and superficial siderosis. Perfusion imaging may provide additional information, but it should not be interpreted in isolation.

Clinicians should avoid treating ARIA and ischemic stroke as mutually exclusive diagnoses. A patient receiving anti-amyloid therapy may have symptomatic ARIA alone, an unrelated ischemic stroke, or cerebral ischemia in the presence of pre-existing or active ARIA. The purpose of multimodal imaging is therefore not merely to assign a diagnostic label. It is to identify hemorrhagic vulnerability, determine whether a causative vascular occlusion is present, and establish which reperfusion options may remain appropriate. This distinction is essential because insufficient caution may expose the patient to hemorrhagic harm, whereas excessive caution may deny effective treatment for a disabling ischemic stroke.

## The reperfusion dilemma

Intravenous thrombolysis presents the most difficult therapeutic decision because treatment is time-sensitive, yet ARIA may increase susceptibility to intracerebral bleeding. Current prescribing information for lecanemab and donanemab advises clinicians to consider whether focal neurological deficits could represent ARIA-E before administering a thrombolytic agent. Appropriate use recommendations are more conservative and advise against intravenous thrombolysis during active anti-amyloid treatment. This caution is supported by reports of fatal intracerebral hemorrhage following thrombolytic administration, including cases in which stroke-like symptoms were subsequently associated with severe ARIA. These reports cannot establish the absolute risk, but they demonstrate that the potential for serious harm is clinically consequential and cannot be dismissed[10].

Anti-amyloid exposure should therefore prompt urgent involvement of a stroke specialist, rapid vascular imaging, and MRI when promptly available and likely to alter management without causing disproportionate delay. Evidence remains insufficient to establish a universally safe interval between the last anti-amyloid dose and thrombolysis or to determine whether MRI without evidence of ARIA eliminates any excess hemorrhagic risk. Decisions should consider the diagnostic confidence in ischemic stroke, severity and potential disability of the deficit, imaging evidence of ARIA or hemorrhage, baseline microhemorrhage burden, and availability of alternative reperfusion strategies[11].

Mechanical thrombectomy should be considered separately from systemic thrombolysis. In a patient with a clearly demonstrated, clinically relevant arterial occlusion, anti-amyloid exposure alone should not automatically preclude endovascular treatment. Mechanical thrombectomy avoids systemic fibrinolysis but does not eliminate hemorrhagic or procedural risk. Established ARIA, infarct extent, baseline cerebrovascular abnormalities, premorbid function, and anticipated procedural benefit remain important considerations. Appropriate caution should reduce avoidable hemorrhagic harm without becoming a reason to withhold potentially effective treatment from every exposed patient.

## Closing the systems gap

Safe decision-making should not rely on a patient with cognitive impairment or a distressed family to recall the name and timing of an anti-amyloid treatment during a stroke code. Anti-amyloid therapy should be incorporated explicitly into emergency medication reconciliation, prehospital handover, stroke checklists, and electronic health record alerts. Patients should receive an emergency treatment card that documents the specific agent, the date of the most recent administration, the treating center, any previous ARIA events, the latest MRI findings, and an urgent contact number.

Centers providing anti-amyloid treatment should also establish shared protocols with emergency medicine, vascular neurology, neuroradiology, and neurointerventional services. These protocols should facilitate rapid access to previous imaging, specify the appropriate MRI sequences, define specialist escalation pathways, and require separate assessments of intravenous thrombolysis and mechanical thrombectomy. Such measures require prospective evaluation and should not be presented as validated risk-reduction interventions. Nevertheless, improving treatment visibility and interdepartmental communication represent a practical response to a foreseeable medication-safety problem[12].

## Conclusion

Anti-amyloid immunotherapy has created a direct clinical intersection between dementia treatment and acute stroke care. Symptomatic ARIA can resemble cerebral ischemia, while unrecognized treatment exposure may complicate time-sensitive reperfusion decisions. Stroke systems should evolve through reliable medication identification, targeted imaging, specialist collaboration, and individualized assessment of intravenous thrombolysis and mechanical thrombectomy. The goal is neither automatic reperfusion nor automatic exclusion, but timely treatment guided by the best-supported diagnosis and the safest appropriate strategy.

## Ethical approval

Not applicable. This editorial did not involve human participants, animals, or identifiable patient data.

## Consent

Not applicable.

## Data Availability

Data sharing is not applicable to this article as no datasets were generated or analyzed during the preparation of this editorial.

## Research registration unique identifying number (UIN)

Not applicable. This article is an editorial and does not involve a clinical trial, systematic review registration, or prospective research registration requiring a unique identifying number.

## References

[1] Alkhalifa AE Al Mokhlf A Ali H. Anti-amyloid monoclonal antibodies for Alzheimer’s disease: evidence, ARIA risk, and precision patient selection. J Pers Med 2025;15:437. 10.3390/jpm15090437 PMC12470750 41003140

[2] Hampel H Elhage A Cho M. Amyloid-related imaging abnormalities (ARIA): radiological, biological, and clinical characteristics. Brain 2023;146:4414–24. 10.1093/brain/awad188 PMC10629981 37280110

[3] van Dyck CH Swanson CJ Aisen P. Lecanemab in early Alzheimer’s disease. N Engl J Med 2023;388:9–21. 10.1056/NEJMoa2212948 36449413

[4] Sims JR Zimmer JA Evans CD. Donanemab in early symptomatic Alzheimer disease: the TRAILBLAZER-ALZ 2 randomized clinical trial. JAMA 2023;330:512–27. 10.1001/jama.2023.13239 PMC10352931 37459141

[5] Greenberg SM Aparicio HJ Furie KL. Vascular neurology considerations for antiamyloid immunotherapy: a science advisory from the American Heart Association. Stroke 2025;56:e30–e38. 10.1161/STR.0000000000000480 39660440

[6] Schreiber CP Kovacik A Bishop J. Amyloid-related imaging abnormalities (ARIA) in the context of Alzheimer’s disease and amyloid-targeting therapies: an introduction for advanced practice providers. Drugs Aging 2025;42:1103–11. 10.1007/s40266-025-01253-x PMC12660453 41042497

[7] Zimmer JA Ardayfio P Wang H. Amyloid-related imaging abnormalities with donanemab in early symptomatic Alzheimer disease: secondary analysis of the TRAILBLAZER-ALZ and ALZ 2 randomized clinical trials. JAMA Neurol 2025;82:461–69. 10.1001/jamaneurol.2025.0065 PMC11894547 40063015

[8] Wang Y Wu W Liu W. Acute stroke diagnosis: diagnostic efficacy of dual-layer spectral computed tomography for non-contrast ischemic sign detection. Quant Imaging Med Surg 2025;15:11094–102. 10.21037/qims-2025-1005 PMC12591734 41209193

[9] Dias BA Bezerra KB Bezerra ASDA. Importance of computed tomography angiography in acute/hyperacute ischemic stroke. Radiol Bras 2021;54:360–66. 10.1590/0100-3984.2020.0168 PMC8630949 34866695

[10] Rabinovici GD Selkoe DJ Schindler SE. Donanemab: appropriate use recommendations. J Prev Alzheimers Dis 2025;12:100150. 10.1016/j.tjpad.2025.100150 PMC12180672 40155270

[11] Bilodeau PA Dickson JR Kozberg MG. The impact of anti-amyloid immunotherapies on stroke care. J Clin Med 2024;13:1245. 10.3390/jcm13051245 PMC10931618 38592119

[12] van Etten ES Mahinrad S Grill JD. Amyloid-related imaging abnormalities (ARIA) in anti-amyloid therapies for Alzheimer’s disease: an update from the Alzheimer’s Association ARIA workgroup. Alzheimers Dement 2026;22:e71361. 10.1002/alz.71361 PMC13099593 42015334

